# Neuro-Immune Modulation Effects of Sacral Nerve Stimulation for Visceral Hypersensitivity in Rats

**DOI:** 10.3389/fnins.2021.645393

**Published:** 2021-07-02

**Authors:** Xue Jin, Payam Gharibani, Jieyun Yin, Jiande D. Z. Chen

**Affiliations:** ^1^Division of Gastroenterology and Hepatology, Department of Medicine, Johns Hopkins University School of Medicine, Baltimore, MD, United States; ^2^Division of Neuroimmunology, Department of Neurology, Johns Hopkins University School of Medicine, Baltimore, MD, United States

**Keywords:** sacral nerve stimulation, visceral hypersensitivity, enteric neurons, mast cells, autonomic functions

## Abstract

**Background:** Visceral hypersensitivity (VH) is one of the underlying pathophysiologies of irritable bowel syndrome. Mast cell overactivation has been found to be one of the main causes of VH. We investigated the effects and mechanisms of actions of sacral nerve stimulation (SNS) on visceral pain in a rodent model of VH.

**Methods:** The VH was established by an intrarectal infusion of AA in 10-day-old pups. Rats were chronically implanted with electrodes for SNS and recording electromyogram (EMG) and electrocardiogram. The acute study was performed in 2-randomized sessions with SNS (14 Hz, 330 μs, 40% motor threshold or MT, 30 min) or sham-SNS. Later on, rats were randomized into SNS/sham-SNS groups and a chronic study was performed with 2 h-daily SNS or sham-SNS for 21 days. Visceromotor reflexes were assessed by abdominal EMG and withdrawal reflex (AWR). Colon tissues were collected to study colonic acetylcholine (ACh), the enteric neurons (ChAT, nNOS, and PGP9.5), mast cells activity [Tryptase, prostaglandins E2 (PGE2), and cyclooxygenases-2 (COX2)] and pain markers [nerve growth factor (NGF) and Sub-P].

**Key Results:** Sacral nerve stimulation significantly improved visceromotor reflexes assessed by the EMG and AWR, compared with sham-SNS. SNS normalized the protein expressions of ChAT and nNOS and regulated mast cells activity by downregulating Tryptase, COX2, and PGE2. Neonatal AA administration upregulated NGF and Sub-P; chronic SNS significantly decreased these pain biomarkers. Concurrently, chronic SNS increased ACh in colon tissues and vagal efferent activity.

**Conclusions:** Sacral nerve stimulation reduces VH in rats and this ameliorating effect might be attributed to the suppression of mast cell overactivation in the colon tissue via the modulation of autonomic nervous system functions.

## Introduction

Visceral pain is a complex disorder sensed by internal organs which is by far the most common type of complaint in gastrointestinal (GI) diseases (Pusceddu and Gareau, 2018). It exerts massive pressure on the healthcare system and impacts the overall quality of life in patients (Hungin et al., 2003; Pusceddu and Gareau, 2018). Most abdominal pain is due to the functional GI disorders, such as irritable bowel syndrome (IBS) and functional dyspepsia (Locke et al., 1997). The most prevalent form of visceral pain is attributed to IBS which affects 3–22% of the general population (Boyce et al., 2000).

Several elements contribute to the pathophysiologies of IBS, such as dysmotility, visceral hypersensitivity (VH), increased intestinal permeability and psychological stress (Whorwell, 2015; Lee and Lee, 2016). However its pathogenesis is still poorly understood, and the treatment outcome is not satisfactory. Low-grade inflammation has been shown to be one of the causes of IBS (Ohman and Simren, 2010) which involves the enteric nervous system (ENS) and immune cells, such as mast cells with direct cross-interactions on each other (De Winter et al., 2012; Karhausen et al., 2013; Jin et al., 2017; Lennon et al., 2018). More attentions have been drawn to the roles of mast cells in GI functions. Mast cells within the GI tract regulate nociception, innate and adaptive immunity, vascular and epithelial permeability, peristalsis, ion secretion, fibrosis and also tissue repair (Bischoff and Kramer, 2007; Boeckxstaens, 2018). Moreover, mast cells can serve as an end effector and release several mediators such as Tryptase/histamine, prostaglandins E2 (PGE2), tumor necrosis alpha (TNF-α) to provoke inflammation, change the permeability (De Winter et al., 2012) and induce VH (Jacob et al., 2005). Hyperplasia and overactivation of the mast cells were shown to induce the main hallmark symptoms of IBS such as abdominal pain, discomfort, bloating and abnormal bowel functions (O’Sullivan et al., 2000; Robles et al., 2019). Compelling evidence has shown that mast cells might be involved in the generation of IBS symptoms, particularly VH (Boeckxstaens, 2018). Targeting mast cells to improve VH has partially exhibited good efficacy in symptom improvement. Accordingly, a more comprehensive approach in treatment is necessary (Camilleri, 2013; Boeckxstaens, 2018).

Electrical stimulation of the peripheral nervous system has been proposed for reducing inflammation and pain in some GI diseases (Locke et al., 1997; Hungin et al., 2003; Whorwell, 2015; Pusceddu and Gareau, 2018), and as such sacral nerve stimulation (SNS) has been approved by the FDA for treating fecal incontinence and overactive bladder (Thin et al., 2013). In a series of animal studies, we have also shown that SNS with appropriate parameters could improve several other GI dysfunctions, such as delayed colonic transit (Peters et al., 2007) and bowel mucosal inflammation (Falletto et al., 2009; Lundby et al., 2011; Jiang et al., 2019b; Tu et al., 2020b). Few studies explored the potential of SNS for VH in animal models (Fassov et al., 2014; Langlois et al., 2015; Jiang et al., 2019a). However, the mode of action needs to be more elucidated. Jiang et al. (2019b) demonstrated that SNS with appropriate parameters could improve VH via the autonomic pathway. While we have also shown that SNS could ameliorate the colonic permeability in animal mode of IBD (Tu et al., 2020b), its mechanisms of action involved in the ameliorating effect of SNS on VH needs further investigation. Thus, in this study, we induced VH by administrating acetic acid (AA) in a neonatal stage, which is a widely accepted IBS animal model. We hypothesized that SNS could ameliorate VH by reducing mucosal mast cell overactivity mediated via the autonomic pathway. The aim of this study was to show that SNS could suppress visceral pain (assessed by visceromotor reflexes) by modulating enteric neurons and regulating mast cells activity in a rodent model of AA-induced VH.

## Materials and Methods

### Animals and Ethics Statement

We used a total number of 20 male Sprague-Dawley (SD) rats in this study in accordance with the guidelines of the Johns Hopkins University for the Care and Use of the laboratory Animals (ACUC’s approved protocol #RA17M292). Six-day-old pups (Charles River, United States) were housed in one cage with one mother and weaned when they were 4 weeks old. The animal room was under controlled conditions with regulated temperature (20–22°C), 50% humidity, and a 12-h light/12-h dark cycle and free access to water and solid food *ad libitum*.

**FIGURE 1:**
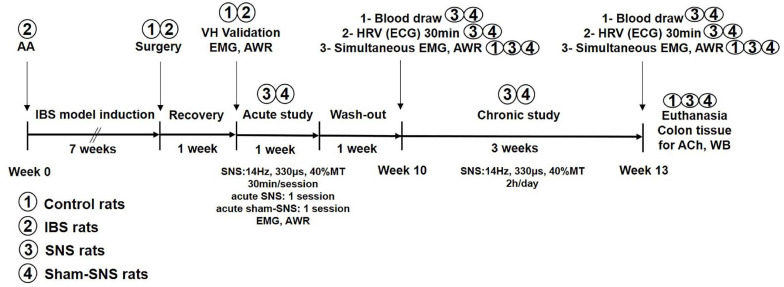
Experimental design and the effects of acute and chronic SNS and sham-SNS on visceral hypersensitivity. EMG and AWR were assessed using colorectal distention test in hypersensitive and control (normal rats). Animal groups are presented by numbers.

The rats were randomized into three groups: SNS group (*n* = 6), sham-SNS group (*n* = 6) and control group (*n* = 8). The animal model of VH was stablished according to previous studies (Al-Chaer et al., 2000; Yin et al., 2010b). Briefly, at the age of 10 days old, the pups in SNS and sham-SNS groups received an infusion of 0.2 ml of 0.5% AA solution in saline (0.09%) into the colon 2 cm from the anus. The control group received only saline using the identical administration method. The rats in the SNS and sham-SNS groups underwent all surgical and experimental procedures outlined in Figure 1. The control rats were only subjected to the electromyogram (EMG)/abdominal withdrawal reflex (AWR) and blood sample collected at Week 10 and Week 13 and colon tissue collection at Week 13.

### Surgical Procedure

The surgical procedure was performed during 8–9 weeks of age for implanting electrodes for three purposes: (Pusceddu and Gareau, 2018) SNS, (Hungin et al., 2003) recording electromyography (EMG) and (Locke et al., 1997) recording electrocardiogram (ECG).

#### SNS Electrodes Implantation

Rats were anesthetized with 2% isoflurane (Abbott Laboratories, Abbott Park, IL, United States) with a 1–2 liter/min oxygen flow. In our previous study, we showed that unilateral but not bilateral SNS improved the inflammation in colitis (Zhang et al., 2020). Therefore, in this study we used unilateral SNS on right sacral nerve of S3 to investigate the possible analgesic effects of SNS. Briefly, as we reported previously (Jiang et al., 2019b; Tu et al., 2020b), a dorsal midline incision was made to expose the right sacral nerve. One pair of electrodes (Cardiac pacing wire, A&E Medical, Farmingdale, NJ, United States) were placed around the right sacral nerve (S3) behind the sacral foramen and fixed by a surgical knot (oval cathode 2–3 mm in length in each electrode). To isolate the exposed wires from the adjacent tissues, we used the dental cement on the wires (Griffin et al., 2011).

#### EMG Electrodes Implantation

An oblique incision was made on the abdominal skin and the tips of two cardiac pacing wires (A&E Medical, Farmingdale, NJ, United States) were implanted at the external oblique muscles in the lower left abdomen to record EMG (Jiang et al., 2019b).

#### ECG Electrodes Implantation

Electrodes were implanted for recording the ECG for measuring heart rate variability (HRV). Briefly, three electrodes (A&E Medical, Farmingdale, NJ, United States) were implanted subcutaneously (two of them under the skin in the chest wall of the chest, and the other one under the skin of abdomen). In order to do that, three skin incisions were made, and the tips of three cardiac pacing wires (A&E Medical, Farmingdale, NJ, United States) were implanted at muscles, underneath the incision sites. All abovementioned electrode connecting wires were subcutaneously tunneled through the back and externalized at the back of neck. The skin incisions were closed with sutures.

Control and sham-SNS animals underwent the same surgical procedures for the placement of electrodes. The rats were given a 7-day recovery following the surgery.

### Experimental Protocols

In this study, the experiments were accomplished at two different steps. In the first step, we validated the success of VH in our animal model. Then, acute and chronic SNS were performed with electrical current produced by a universal pulse generator (Model DS8000, World Precision Instruments, Sarasota, FL, United States) through the chronically implanted SNS electrodes. The experimental design is shown in Figure 1 and described as follows.

### Measurement of Visceromotor Reflexes

The EMG of external oblique muscles as well as AWR were assessed in response to the colorectal distention (CRD) for assessing visceromotor reflex, as previously reported (Al-Chaer et al., 2000; Jiang et al., 2019b).

#### Recording and Measurement of EMG

Under a mild anesthesia with 1% isoflurane, a 5 cm-flexible balloon (made from a finger of a surgical glove) attached to a Tygon tube was inserted into the rat anus and advanced for about 8 cm from anal verge to the descending colon and held in place by taping the tube to the tail. Rats were allowed to adapt for 30 min in a small Lucite cubicle (20 × 8 × 8 cm) before the CRD test. An EMG100C amplifier (MP100, BIOPAC Systems, Inc., Santa Barbara, CA, United States) was used to record the EMG signal during the whole process (before, during and after CRD). To test the visceromotor reflex via the EMG, the balloon was rapidly inflated to a constant pressure of 20, 40, 60, and 80 mmHg determined by a sphygmomanometer. Each distention lasted for 20 s and was followed by a 2 min rest (pressure at 0 mmHg). Then the distention was repeated for one more time. The EMG signals were amplified and digitized at a frequency of 2000 Hz using the accompanying software (ACQKNOWLEDGE, BIOPAC System, Inc., Santa Barbara, CA, United States). The area under the curve (AUC) of the EMG signal during each 20 s of distention was calculated using an in-house written computer program. Meanwhile, the AUC of the EMG 20 s before, 20 s during and 20 s after each distention were calculated. The change of the EMG at each distention pressure was calculated by the AUC during the distention subtracting the mean value of the AUC before each distention. The final results were presented by the mean value of the EMG change in 2 repeated sessions (Yin et al., 2010b).

#### Recording and Measurement of AWR

The AWR was assessed by two colleagues who were blinded to the study protocol. It was assessed simultaneously during the graded CRD for the EMG recording and scored from 1 to 4 as follows: 1: no behavioral response to CRD; 2: contraction of abdominal muscles; 3: lifting of abdomen; and 4: body arching and lifting of pelvic structures (Al-Chaer et al., 2000).

### Assessments of Autonomic Functions

#### Recording and Measurement of Heart Rate Variability

As shown in Figure 1, the ECG was recorded before the initiation of chronic SNS (week 10) and at the end of the treatment (week 13) for 30 min period in the fasting state using a special one-channel amplifier with a cutoff frequency of 100 Hz (Fetrode Amplifier, model 2283 ft/I, UFI, Morro Bay, CA, United States). An HRV signal was derived using a previously validated software (Yin et al., 2010a) by identifying R waves, calculating R-R intervals, interpolating the R-R interval data and sampling at a frequency of 8 Hz. The overall power spectrum of the HRV signal was then calculated and the total power in each of 2 frequency sub-bands was assessed, including low frequency (LF) and high frequency (HF) (Yin et al., 2010a). The power in the LF band (0.3–0.8 Hz) represents mainly sympathetic activity and the power in the HF band (0.8–4.0 Hz) stands purely for vagal activity (Tu et al., 2020c). The ratio LF/HF reflects the balance between sympathetic activity and vagal activity.

#### Measurements of Endocrine Hormones, Norepinephrine and Pancreatic Polypeptide

Blood samples were drawn in the fasting state before the initiation of chronic SNS (week 10) and at the end of the treatment (week 13, see Figure 1). Plasma pancreatic polypeptide (PP) (CSB-E12747r, CUSABIO, United States) and norepinephrine (NE) (ab47831, Abcam, Cambridge, United Kingdom) were measured by ELISA, according to the manufacturer’s protocols.

#### Measurement of Acetylcholine Release in Colon

At the end of experiment, colonic acetylcholine (ACh) was measured as described previously (Tu et al., 2020b) in all rats, including the control rats. Briefly, the colonic tissues were dissected, and the mucosal layer was scarped on an ice-cold dish with clean tools as quickly as possible to prevent degradation by endogenous proteases. Tissue samples were homogenized in 0.01 M PBS using a glass tissue homogenizer and centrifuged at 5,000 × *g* for 10 min. The resulting supernatants were collected, and the protein concentrations were measured using the Bradford Protein Assay (Bio-Rad). The content of ACh in colonic tissue samples was assessed using an ACh assay kit (Abbexa Ltd., Catalog No abx051982) according to the manufacturer’s protocol (Sun et al., 2013).

### Validation of Animal Model of Visceral Hypersensitivity

This experiment (marked as “VH validation” in Figure 1) was performed to validate if the VH model was induced successfully. The AA-treated rats (*n* = 12) and saline-treated controls (*n* = 8) were tested 8 weeks after AA or saline administration. The EMG and AWR were recorded and analyzed at baseline and during CRD at different pressures (20, 40, 60, and 80 mmHg). The AWR was graded at the same time by two colleagues who were blinded to the study protocols.

### Effect of Acute and Chronic SNS on Visceral Hypersensitivity

In this experiment, we investigated if acute SNS was able to improve the visceromotor reflexes in AA-treated rats and then, how chronic SNS might have ameliorating effects in the AA-treated rats. During week 9 (marked as “acute study” in Figure 1), all 12 AA-treated rats were subjected to two randomized sessions of SNS or sham-SNS with an interval of 3 days. In each session, the EMG and AWR were simultaneously recorded immediately after 30-min SNS (14 Hz, 330 μs, 40% motor threshold or MT) (Jiang et al., 2019b) or sham-SNS (identical with SNS except there was no actual stimulation). The MT was defined as the lowest level of SNS capable of causing a contraction in the tail muscle that can be seen by bare eyes (Tu et al., 2020b).

In the chronic study, the 12 AA-treated rats were randomized into SNS group (*n* = 6) and sham-SNS group (*n* = 6). The SNS group (*n* = 6) received daily SNS (14 Hz, 330 μs, 40%MT, 2 h) for 3 weeks, whereas, the sham-SNS group (*n* = 6) received daily sham-SNS (identical with SNS except there was no actual stimulation). Following measurements were made sequentially (see Figure 1) in the fasting state before and after the chronic treatment: after, the collection of blood samples from tail vein, a 30-min ECG recording (for HRV) was made and then the EMG and AWR responses to CRD were simultaneously assessed. The control group (*n* = 8) received no treatment.

At the end of the entire study, all animals (including controls) were sacrificed, and colon tissues were collected for the mechanistic assessment.

### Mechanisms of Chronic SNS on Visceral Hypersensitivity

At the end of the study, all rats terminated by an overdose of isoflurane (adjusting the isoflurane flow rate to 5% until breathing stopped) and opening the abdominal cavity and chest for tissue collection. Colon tissue samples (middle third: 8–12 cm proximal to the anus as total length of 4 cm) were collected for assessing key biomarkers in ENS (PGP9.5, ChAT, and nNOS) as well as mast cells [Tryptase, PGE2, and cyclooxygenases-2 (COX2)] and pain markers [nerve growth factor (NGF) and Substance-P] by western blot (WB).

#### Western Blot Analysis

The colon tissue samples were lysed in a RIPA buffer containing a 2% phosphatase-inhibitor (Thermo Fisher Scientific, Waltham, MA, United States) and a 1% mammalian-protease inhibitor (Sigma-Aldrich). 50 μg of extracted proteins were run a single track of 10% sodium dodecyl sulfate–polyacrylamide gel electrophoresis (SDS-PAGE), and the separated proteins were transferred electrophoretically onto cellulose membranes. The membranes were blocked in 5% non-fat dry milk for 60 min, and then incubated with primary antibodies against nNOS (1:1000; Cell Signaling, Boston, MA, United States), Sub-P (1:100; Novus biologicals, Centennial, CO, United States), ChAT (1:100), NGF (1:200), COX2 (1:300), Tryptase (1:500), PGP9.5 (1:1000) and GAPDH (1:10,000) (all Abcam, Cambridge, United Kingdom), and PGE2 polyclonal antibody (1 : 200; Bioss Inc., Woburn, MA, United States) (Chia et al., 2017) overnight at 4°C. The membranes were washed 3× with TBS-T (TBS mixed with 0.1% Tween-20) and then, they were incubated with ECL AP-conjugated anti-rabbit/mouse IgG (1:3,000; GE Healthcare, United Kingdom) for 90 min in room temperature. Quantitative western blot results were obtained by densitometric analysis using image processing and analysis in Java (Image J, NIH, Bethesda, MD, United States). The percent change of relative intensity was calculated against control samples.

### Statistical Analysis

All results are expressed as mean ± SE. Differences between multiple groups were evaluated by Analysis of variance (One-way or Two-way ANOVA). For EMG and AWR, Two-way ANOVA was used to compare the difference between baseline and animal model of IBS or after treatments. While One-way ANOVA was used for HRV and ACh, Student’s unpaired *t*-test was performed for NE/PP and western-blot. *P* value ≤ 0.05 was considered significant. All statistical analysis was performed by using GraphPad Prism 8.3.

## Results

### Acetic Acid Induced Visceral Hypersensitivity

**FIGURE 2:**
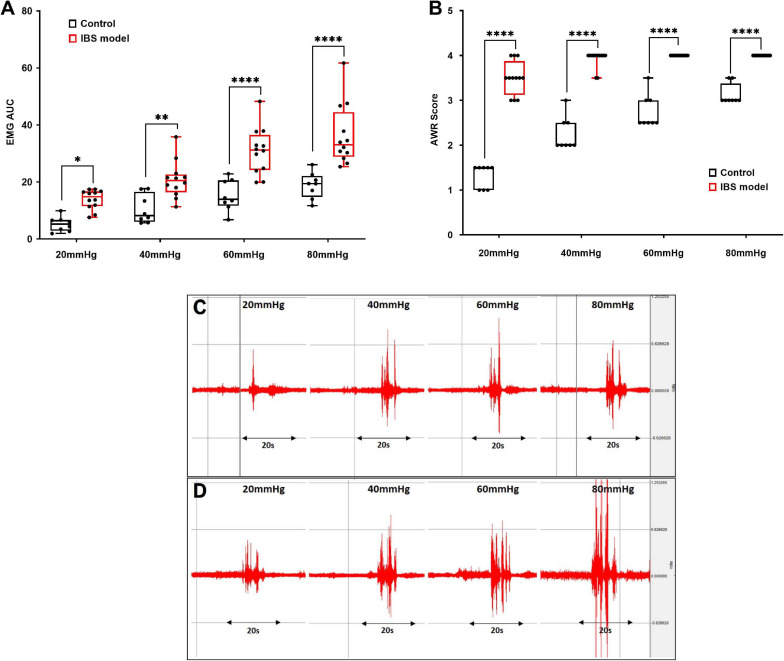
Validation of animal model of visceral hypersensitivity (IBS model). EMG and AWR were assessed using colorectal distention test in hypersensitive (*n* = 12) and control (normal) rats (*n* = 8). **(A,C,D)** EMG in acetic acid-treated rats were significantly higher at 20, 40, 60, and 80 mmHg than control (*p < 0.03, **p < 0.005, ****p < 0.0001; Two-way ANOVA, Bonferroni). **(C,D)** Representative EMG examples of control **(C)** and IBS **(D)** rats at 20, 40, 60, and 80 mmHg. Each CRD was carried out for 20 s. **(B)** AWR scores in acetic acid-treated rats were significantly higher at different colorectal distention pressures compared to the control. Values were represented as the means ± SE, (****p < 0.0001; Two-way ANOVA, Bonferroni). IBS: inflammatory bowel disease; EMG: electromyogram; AWR: abdominal withdraw reflex; AUC: area under the curve; CRD: colorectal distention.

In order to carry out the SNS experiment, firstly we validated if VH was induced successfully in rats treated with AA. Compared with the control (*n* = 8) rats, the abdominal visceromotor (EMG) and AWR were increased significantly in the AA-treated rats (*n* = 12) responding to CRD. The area under the curve of EMG in the AA-treated rats was increased from 5.32 ± 0.92 to 13.92 ± 0.1; 10.44 ± 1.7 to 20.8 ± 1.8; 15.3 ± 1.9 to 30.8 ± 2.3; and 18.86 ± 1.6 to 36.32 ± 3.08 compared to the control at 20, 40, 60, and 80 mmHg CRD, respectively (Figures 2A,C,D, *p* < 0.03; Two-way ANOVA, Bonferroni). AWR scores in the AA-treated rats were increased from 1.31 ± 0.09 to 3.5 ± 0.1; 2.25 ± 0.13 to 3.9 ± 0.05; 2.75 ± 0.12 to 4.0 ± 0.0; and 3.12 ± 0.08 to 4.0 ± 0.0 compared to the control rats at 20, 40, 60, and 80 mmHg CRD, respectively (Figure 2B, *p* < 0.001; Two-way ANOVA, Bonferroni). Thus, it was confirmed that VH was induced successfully in AA-treated rats.

### Ameliorating Effects of Acute SNS on Visceromotor Reflexes

**FIGURE 3:**
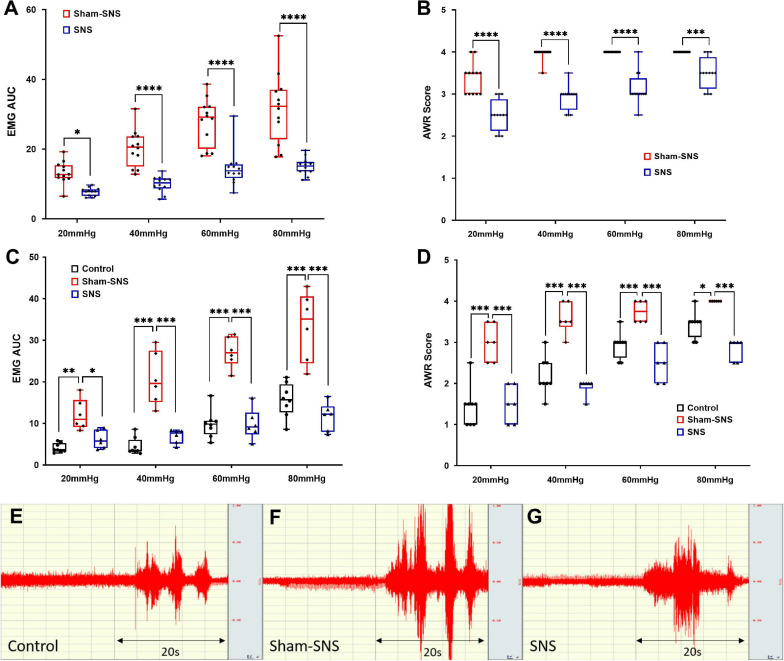
The effects of acute and chronic SNS and sham-SNS on visceral hypersensitivity. EMG and AWR were assessed using colorectal distention test in hypersensitive and control (normal rats). **(A,B)** Effects of acute SNS (14 Hz, 330 μs, 40% motor threshold, 30 min) on EMG and AWR of SNS and sham-SNS groups (*n* = 12). In animal model of hypersensitivity, EMG and AWR were significantly lower in SNS-treated rats compared to sham-SNS (*p < 0.05, ***p < 0.001, ****p < 0.0001; EMG: Two-way ANOVA, Bonferroni). **(C,D)** Effects of chronic SNS (14 Hz, 330 μs, 40% motor threshold, 3 weeks, 2 h/day, *n* = 6) on EMG and AWR of SNS and sham-SNS groups (*n* = 6). Chronic SNS significantly reduced the EMG and AWR compared to sham-SNS and normalized the values comparable to the control (*n* = 8) (*p < 0.02, **p < 0.002, ***p < 0.003, ****p < 0.0001; Two-Way ANOVA, Tukey). **(E–G)** EMG tracing of CRD at 80 mmHg for 20 s in control, sham-SNS and SNS groups following chronic treatment. Values were represented as the means ± SE. AA, acetic acid; SNS, sacral nerve stimulation; HRV, heart rate variability; WB, western blot; IHC, immunohistochemistry; EMG, electromyogram; AUC, area under the curve; AWR, abdominal withdraw reflex; CRD, colorectal distention.

As shown in Figure 1, following the validation of our animal model of VH, we tested the effects of acute SNS (14 Hz, 330 μs, 40%MT, 30 min) or sham-SNS on EMG and AWR during CRD. Acute SNS significantly reduced EMG and AWR in AA-treated rats compared to the sham-SNS. SNS was able to decrease the area under the curve of EMG by 41.81% (13.26 ± 0.91 to 7.71 ± 0.34), 51.0% (20.29 ± 1.52 to 9.95 ± 0.67), 48.12% (27.91 ± 1.93 to 14.48 ± 1.53), and 52.6% (31.7 ± 2.88 to 15.02 ± 0.71) at 20, 40, 60, and 80 mmHg CRD, respectively compared to the sham-SNS (Figure 3A, *n* = 12, *p* < 0.05; Two-way ANOVA, Bonferroni). Similarly, AWR scores were also decreased by 23.66% (3.37 ± 0.1 to 2.5 ± 0.1), 26.3% (3.95 ± 0.04 to 2.91 ± 0.08), 21.87% (4.0 ± 0.0 to 3.12 ± 0.1), and 12.5% (4.0 ± 0.0 to 3.5 ± 0.1) at 20, 40, 60, and 80 mmHg CRD, respectively compared to the sham-SNS (Figure 3B, *n* = 12, *p* < 0.001; Two-way ANOVA, Bonferroni). However, it can be seen from the figure that the AWR was a less sensitive measure of visceromotor reflexes as its score almost reached the maximum at CRD of 40 mmHg.

### Mechanisms of Chronic SNS on Visceral Hypersensitivity

Chronis SNS was performed for 2 h/day for a period of 3 weeks with the same parameters. The EMG and AWR were recorded during CRD before and after the chronic SNS. Meanwhile, we collected blood and recorded the ECG to assess the effects of chronic SNS on the autonomic system function (experimental design: Figure 1).

#### Chronic SNS Ameliorated Visceromotor Reflexes in Visceral Hypersensitive Rats

Compared to the sham-SNS, chronic SNS decreased the area under the curve of EMG by 49.17% (12.1 ± 1.57 to 6.15 ± 0.88), 66.5% (20.73 ± 2.59 to 6.94 ± 0.69), 63.76% (27.18 ± 1.5 to 9.85 ± 1.5), and 65.08% (33.3 ± 3.39 to 11.64 ± 1.38) at 20, 40, 60, and 80 mmHg CRD, respectively (Figures 3C,E–G, *p* < 0.04; Two-way ANOVA, Tukey). EMG in sham-SNS group but not in SNS was significantly higher compared with control group. AWR scores were also reduced by 50.0% (3 ± 0.18 to 1.5 ± 0.18), 46.51% (3.58 ± 0.15 to 1.92 ± 0.08), 33.3% (3.75 ± 0.11 to 2.5 ± 0.18), and 29.16% (4 ± 0 to 2.83 ± 0.1) at 20, 40, 60, and 80 mmHg CRD, respectively compared to the sham-SNS (Figure 3D, *p* < 0.02; Two-way ANOVA, Tukey). Interestingly, chronic SNS was able to normalize both EMG and AWR at different pressure of CRD, comparable to the normal rats (control).

#### Chronic SNS Improved the Autonomic Nervous System Functions

**FIGURE 4:**
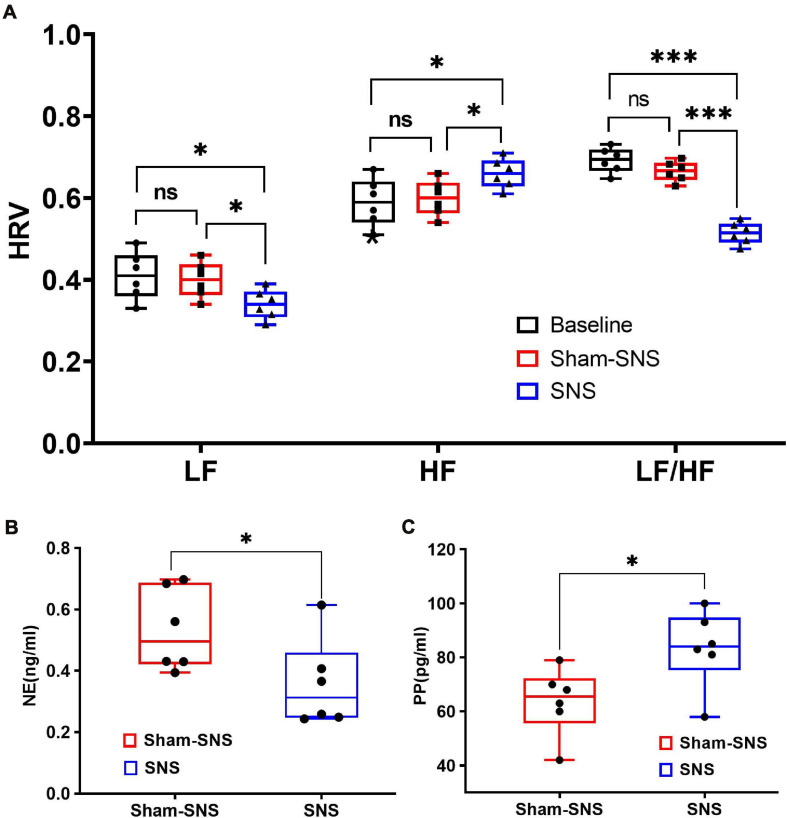
Effects of chronic SNS and sham-SNS on autonomic functions. **(A)** Upper panel shows the effects of chronic SNS and sham-SNS on vagal and sympathetic activities of visceral hypersensitive rats. HF and LF were measured by heart rate variability derived from ECG recording. HF (0.8–4.0 Hz) represents vagal activity, while LF (0.3–0.8 Hz) represents sympathetic activity. LF/HF ratio reflects the balance between sympathetic and vagal activity. Chronic SNS (but not sham-SNS) for 2 h/day for 14 days, significantly increased vagal activity (HF) and decreased sympathetic activity (LF) and the balance between sympathetic and parasympathetic activity (LF/HF) (ns: not significant, *p < 0.05, ***p < 0.001; One-Way ANOVA, Tukey). The unit for both LF and HF was the decibel. **(B,C)** Lower panel show the effects of chronic SNS on endocrine hormones NE and PP before and after treatment. **(B)** Represents the endocrine hormone NE showing the level of sympathetic activities in SNS and sham-SNS groups. **(C)** Represents the endocrine hormone PP showing the level of vagal activities in SNS and sham-SNS groups. NE and PP dramatically changed after 3-week SNS treatment compared to sham-SNS (all significantly changed; *p < 0.05; Student *t*-test). Values were represented as the means ± SE (*n* = 6). Baseline: represents HRV before treatment initiation. SNS, sacral nerve stimulation; HRV, heart rate variability; HF, high frequency; LF, low frequency; NE, norepinephrine; PP, pancreatic polypeptide.

Autonomic nervous system functions were measured via assessing the HRV derived from the ECG as well as measuring the release of endocrine hormones of NE and PP in the blood drawn from vein (Figure 4).

##### The heart rate variability derived from ECG

As shown in Figure 4A, following 3-week treatment, SNS could significantly decrease the sympathetic activity (LF) from 0.41 ± 0.05 to 0.34 ± 0.04 (*p* < 0.05) compared to the sham-SNS. On the other hand, chronic SNS increased the vagal activity (HF) from 0.59 ± 0.05 to 0.66 ± 0.04 (*p* < 0.05). LF and HF showed no changes in value in our animal model of VH compared to the control (*p* > 0.05). 3-week chronic SNS, could significantly reduce LF/HF value compared to the control and sham-SNS groups (SNS: 0.51 ± 0.08, sham-SNS: 0.66 ± 0.024, baseline: 0.69 ± 0.03; *p* < 0.001; One-Way ANOVA, Tukey).

##### Endocrine hormones of NE and PP

Sympathetic (norepinephrine, NE) and vagal (pancreatic polypeptide, PP) endocrine hormones, were measured by Elisa, before and after the chronic treatment in both SNS and sham-SNS groups. As shown in Figures 4B,C, while plasma NE was significantly decreased compared to the sham-SNS, PP conversely increased after the 3-week SNS treatment. While SNS notably increased the PP value from 63.6 ± 5.09 pg/ml in sham-SNS to 83.33 ± 5.83 pg/ml in SNS treatment group (*p* < 0.03; Student *t*-test), plasma NE was reduced from 0.53 ± 0.05 ng/ml in sham-SNS to 0.36 ± 0.059 ng/ml in SNS treatment group (*p* < 0.05; Student *t*-test).

##### ACh release in colon tissue

**FIGURE 5:**
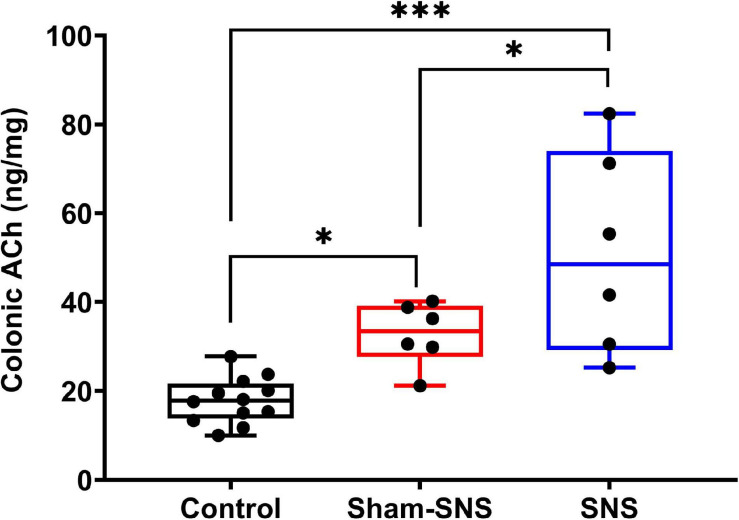
Effects of chronic SNS and sham-SNS on colonic ACh release. ACh in colon tissues was significantly increased in SNS group. Values were represented as the means ± SE (control: *n* = 6, SNS and sham-SNS: *n* = 6). SNS, sacral nerve stimulation (*p < 0.05, ***p < 0.0001; One-Way ANOVA, Tukey).

Acetylcholine is known to play an important role in the cholinergic anti-inflammatory pathway. AA administration induced endogenous increase of ACh in colon tissue (sham-SNS: 32.48 ± 2.80) compared with the control group (normal rats) (17.80 ± 1.48) (*p* < 0.05; One-Way ANOVA, Tukey). Despite this, the SNS treatment increased further the ACh release in SNS group (51.06 ± 9.20) (*p* = 0.04, vs. sham-SNS, respectively; One-Way ANOVA, Tukey) (Figure 5).

#### Restoring Effects of Chronic SNS on Enteric Nervous System Functions

After a 3-week SNS or sham-SNS treatment, rats were sacrificed, and the middle third of the colon was harvested to investigate the treatment impacts on neuronal as well as pain related biomarkers. Normal rats were used as control.

##### ENS markers: ChAT, nNOS, and PGP9.5

**FIGURE 6:**
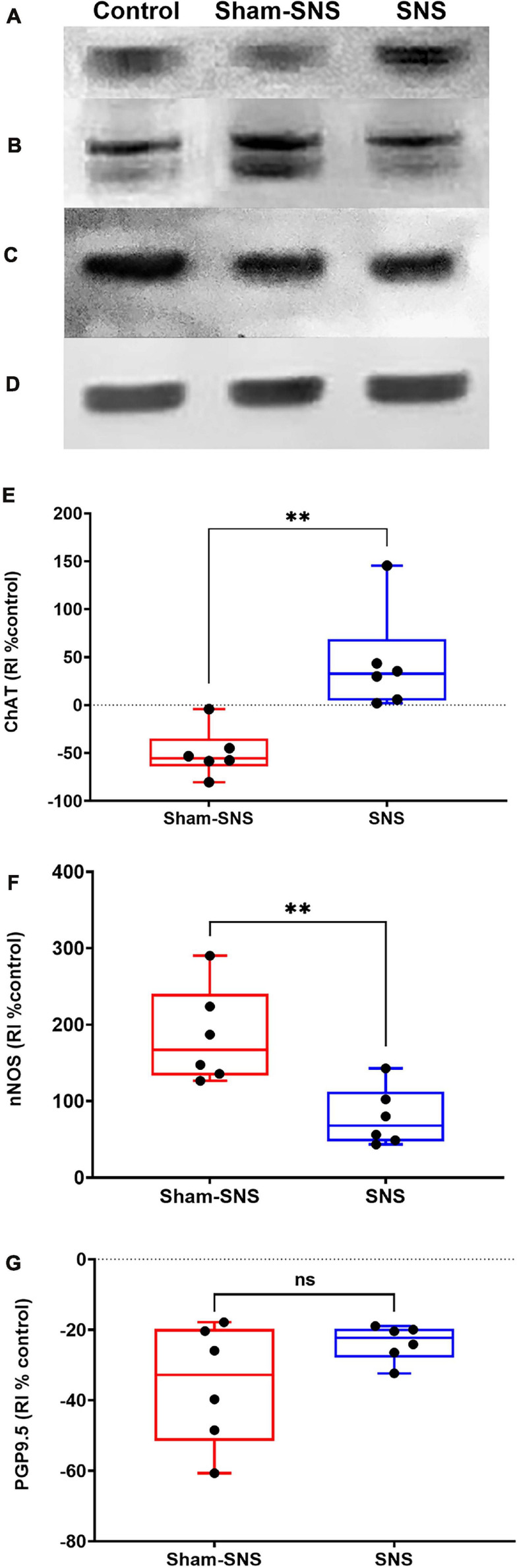
Effects of chronic SNS and sham-SNS on protein expression of ChAT **(A,E)**, nNOS **(B,F)**, PGP9.5 **(C,G)**, and GAPDH **(D)** in middle third of colon of visceral hypersensitive rats in comparison with the control (normal rats). **(A)** Illustrates ChAT immunoblot (72 kDa), while panel **(E)** displays the percent change of protein expression of ChAT in colon tissue compared to control group. SNS markedly upregulated ChAT protein expression in colon tissue after 3 weeks treatment. Panel **(B)** shows nNOS immunoblot (170 kDa), while panel **(F)** represents the percent change of protein expression of nNOS in colon tissue compared to control group. nNOS that was upregulated in sham-SNS group was downregulated after a 3-week SNS. Panel **(C)** show PGP9.5 immunoblot (25 kDa), while panel **(G)** illustrates the percent change of protein expression of PGP9.5 in colon tissue compared to control group. PGP9.5 protein expression did not alter by 3-week SNS. Values were represented as the means ± SE (*n* = 6) as percentage of relative intensity normalized to internal control (GAPDH, 36 kDa). SNS, sacral nerve stimulation, RI, relative intensity **p < 0.01; Student hbox*t*-test).

Figure 6 shows the protein expression of ChAT, nNOS, and PGP9.5 in colon tissues of all treatment groups. The neonatal AA treatment induced adverse effects in the protein expressions of ChAT, nNOS, and PGP9.5 in the rats that were not treated with chronic SNS. While the AA treatment decreased the protein expression of ChAT by 50.29% in the sham-SNS group (*p* = 0.009 vs. control); SNS was able to normalize the expressions of this protein (*p* = 0.002 vs. sham-SNS; *p* > 0.05 vs. control) (Figures 6A,E). Conversely, nNOS protein expression that was upregulated more that 2-folds in the sham-SNS group (*p* = 0.0002 vs. control), was significantly diminished by the SNS treatment (*p* = 0.01 vs. sham-SNS; Student *t*-test) (Figures 6B,F). On the other hand, as shown in Figures 6C,G, the protein expression of PGP9.5 was downregulated by almost 33.1% in the sham-SNS group compared to the control (*p* = 0.046; Student *t*-test), however, chronic SNS was not able to alter its expression after a 3-week treatment (Figures 6C,G).

#### Molecular Mechanisms of SNS on Visceral Sensation

##### Mast cell markers: PGE2, COX2, and Tryptase

**FIGURE 7:**
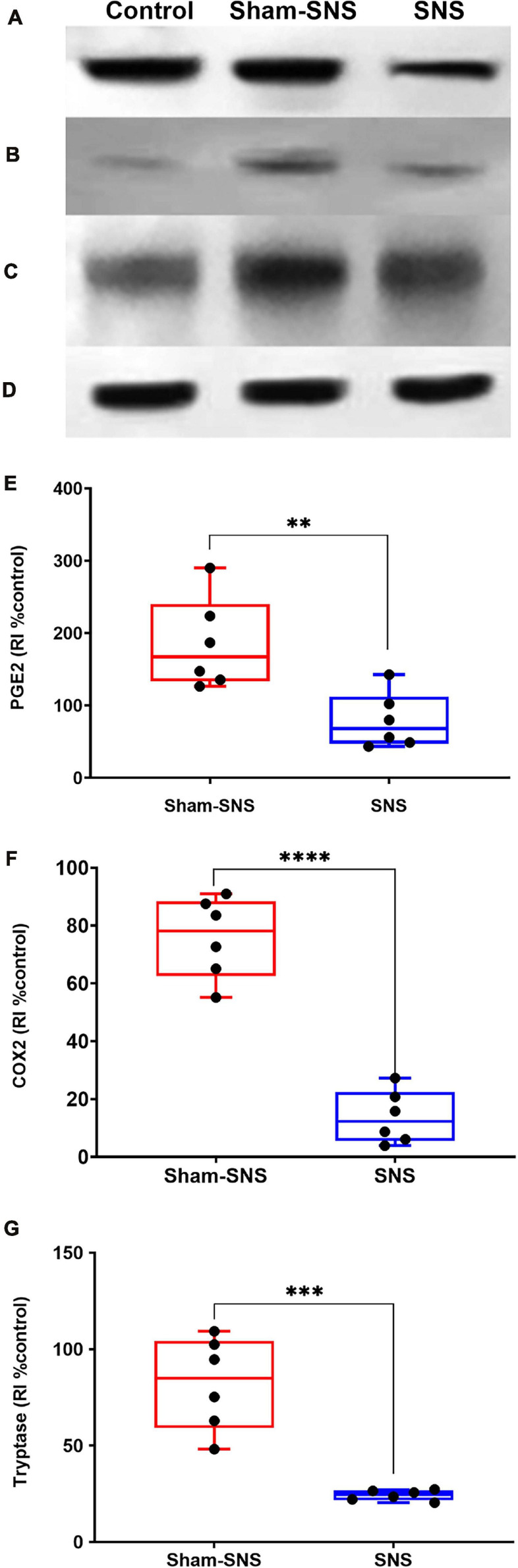
Effects of chronic SNS and sham-SNS on protein expression of PGE2 **(A,E)**, COX2 **(B,F)**, Tryptase **(C,G)**, and GAPDH **(D)** in middle third of colon of visceral hypersensitive rats in comparison with the control (normal rats). Panel **(A)** shows PGE2 immunoblot (42 kDa) and panel **(E)** displays the percent change of protein expression of PGE2 in colon tissue compared to control group. SNS markedly downregulated PGE2 protein expression in colon tissue after 3 weeks treatment. Panel **(B)** shows COX2 immunoblot (75 kDa) and panel **(F)** exhibits the percent change of protein expression of COX2 in colon tissue compared to control group. COX2 that was upregulated in sham-SNS group was downregulated after a 3-week SNS. Panel **(C)** shows Tryptase immunoblot (52 kDa) and panel **(G)** presents the percent change of protein expression of Tryptase in colon tissue compared to control group. Tryptase protein that was upregulated in sham-SNS group was down-regulated after a 3-week SNS. Values were represented as the means ± SE (*n* = 6) as percentage of relative intensity to normalized to internal control (GAPDH, 36 kDa). SNS, sacral nerve stimulation, RI, relative intensity (**p < 0.01, ***p < 0.001, ****p < 0.0001; Student *t*-test).

As shown in Figure 7, the neonatal AA treatment upregulated the protein expressions of PGE2, COX2 and Tryptase in the colon tissues in sham-SNS group compared to the control (all *p* ≤ 0.03; One-Way ANOVA, Tukey). Interestingly, SNS was able to normalize these protein expressions comparable to the control (PGE2: *p* = 0.016, COX2: *p* = 0.021, Tryptase: *p* = 0.03 vs. sham-SNS, respectively; Student *t*-test).

##### Pain markers: NGF and substance-P

**FIGURE 8:**
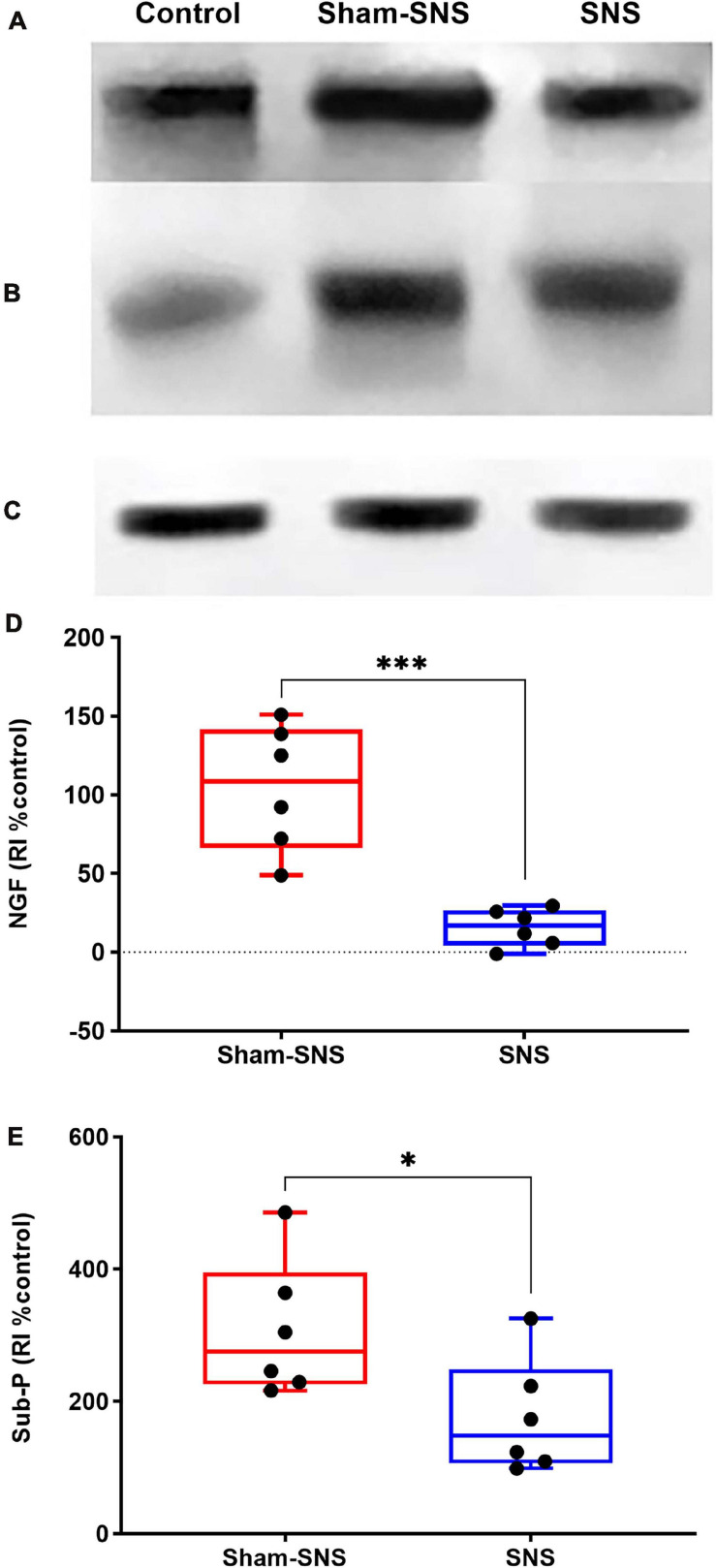
Effects of chronic SNS and sham-SNS on protein expression of NGF **(A,D)**, Sub-P **(B,E)**, and GAPDH **(C)** in middle third of colon of visceral hypersensitive rats in comparison with the control (normal rats). Panel **(A)** shows NGF immunoblot (140 kDa) and panel **(D)** represents the percent change of protein expression of NGF in colon tissue compared to control group. SNS markedly downregulated NGF protein expression in colon tissue after 3 weeks treatment. Panel **(B)** shows Sub-P immunoblot (50 kDa) and panel **(E)** displays the percent change of protein expression of Sub-P in colon tissue compared to control group. Sub-P that was upregulated in sham-SNS group was downregulated after a 3-week SNS. Values were represented as the means ± SE (*n* = 6) as percentage of relative intensity normalized to internal control (GAPDH, 36 kDa). NGF, nerve growth factor; Sub-P, substance-P; SNS, sacral nerve stimulation, RI, relative intensity (*p < 0.05, ***p < 0.001; Student *t*-test).

As shown in Figure 8, the neonatal AA treatment upregulated the protein expressions of NGF and Sub-P more than two-folds in the colon tissues in sham-SNS group compared to the control (both *p* ≤ 0.003; One-Way ANOVA, Tukey). Interestingly, SNS was able to downregulate NGF and Sub-P protein expressions compared to the sham-SNS (*p* = 0.007 and *p* = 0.03, respectively; Student *t*-test).

## Discussion

**FIGURE 9:**
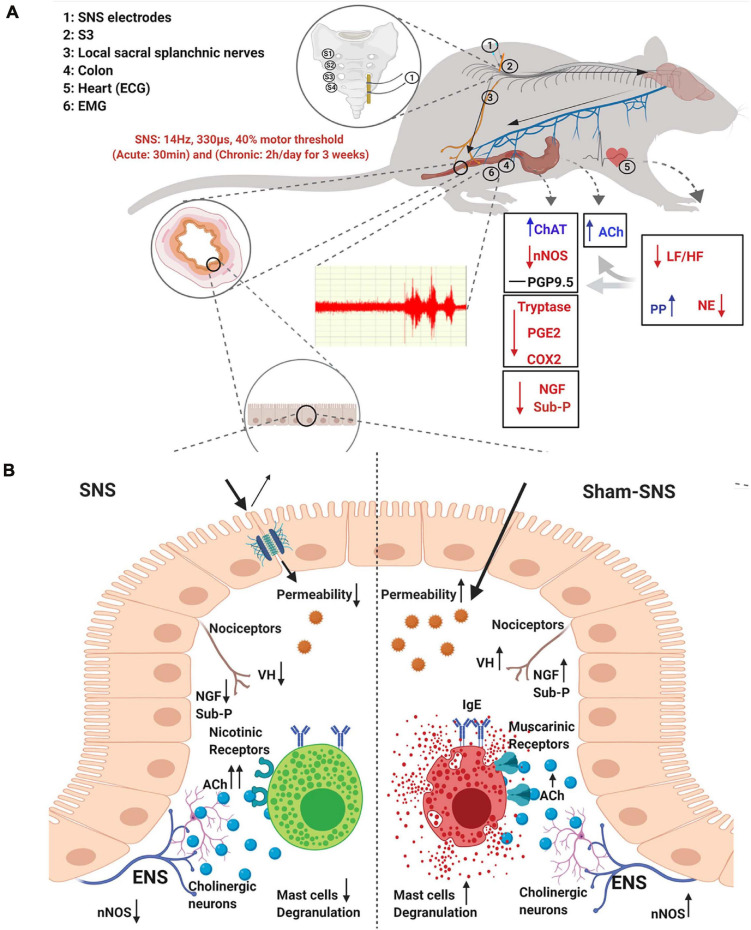
Schematic figure of the possible mechanism of effects of SNS on VH. **(A)** Possible extrinsic afferent circuitry. **(B)** Possible intrinsic mechanism.

In this study, we found that (1) SNS was able to improve the sympathovagal balance and the endocrine hormones, PP and NE, and through that (2) SNS reduced VH possibly by modulating the interactions between the enteric neurons and mast cells as: (i) SNS could regulate the enteric neurons to increase ACh release as well as upregulating the ratio of ChAT protein expression over nNOS; (ii) SNS reduced the mast cells overactivation by the normalization of the protein expressions of Tryptase, PGE2 and COX2; (iii) SNS downregulated the pain markers of NGF and Sub-P; and finally (3) SNS improved the treatment outcome verified by reduced visceromotor reflex (EMG and AWR) during CRD. To the best of our knowledge, this was the first study to report that SNS with appropriate parameters was effective in suppressing VH by modulating the ENS and mast cells via the autonomic pathway in visceral hypersensitive rats. The schematic figure shown in Figure 9 illustrates the above mechanisms involved in the analgesic effect of SNS.

While IBS has been treated with a variety of medications, the effectiveness of commonly used clinical medications is not always adequate due to the complexity of its symptoms and etiology. Moreover, lack of effective treatments for IBS casts a large socioeconomic cost (Enck et al., 2016; Grundy et al., 2019). Traditional analgesics, such as non-steroidal anti-inflammatory drugs (NSAIDs) and opioids, are unsuitable for treatment due to potentially severe side effects, such as increasing dependency and tolerance, and/or inducing constipation (Grundy et al., 2019). Electroacupuncture has been shown in clinical trials to effectively improve functional GI disorders including IBS and functional dyspepsia (Ouyang and Chen, 2004; Takahashi, 2006). While electroacupuncture compared to other treatments is considered a safer alternative with less side-effects, its mechanisms of actions on VH have not been well-studied.

In contrast to somatic nociception, the gut nociception is more complex due to the presence of two extrinsic innervation systems (vagal and spinal), intertwined with intrinsic innervation structure, called the ENS (Knowles and Aziz, 2009). The colon and rectum are innervated by spinal sensory afferents from two distinct regions: the lumbar splanchnic and sacral pelvic nerves pathways (Grundy et al., 2019). The innervation for nociception is contributing to receiving stimulus in the mucosa/muscular layers and then to transducing the pain to the dorsal horn of spinal cord via dorsal root ganglia of T10–L2 and L5–S1 (Chichlowski and Rudolph, 2015). Then the pain message is relayed by spinothalamic and spinoparabrachial pathway to the supraspinal centers (Al-Chaer and Traub, 2002; Grundy et al., 2019) at the cingulate cortex, medial thalamus, amygdala, hypothalamus, periaqueductal gray, and the solitary tract (Jones et al., 2006). While these centers are important components of the brain for pain perception, spinal afferent has been extensively studied in basic and clinical research as a classical method for visceral pain treatment by the spinal cord stimulation (Coffin et al., 2004; Yampolsky et al., 2012). In this study, our focus was on the ameliorating effects of SNS on VH that might be mediated by the regulation of the autonomic nervous functions on the colon. This effect might take place via two pathways: the local sacral splanchnic nerve pathway and the sacral afferent-brain stem-vagal efferent pathway. In fact, in a previous study we showed that there was a possible sacral afferent-brain stem-vagal efferent pathway that could transmit SNS to the brain and then via the vagal efferents to the colon tissue (Tu et al., 2020b).

It was reported that the autonomic functions could play an important role in visceromotor reflex and VH (Jiang et al., 2019b; Tu et al., 2020a). As vagus nerve has been more and more validated to have a major role in regulating visceral sensitivity (Swartz and Holmes, 2014; Inoue et al., 2016; Tu et al., 2020a), the vagus nerve neuromodulation is closely pursued in the treatment of visceral pain (Chen et al., 2008). From one hand, suppression of sympathetic activity by SNS might not only block the spinal afferent signal carrying pain to the brain, but also desensitize the spinal sensory pathway in both nociceptors and dorsal root ganglion levels (Xia et al., 2011; He et al., 2017). On the other hand, SNS might take actions via both vagal nerve and local splanchnic nerves to send a signal to the colon and to suppress neurotransmitters associated with VH, such as NGF and Sub-P (Coffin et al., 2004; Chen et al., 2008; Ohman and Simren, 2010; Li et al., 2016) and/or special cells highly associated with VH such as mast cells (Bischoff and Kramer, 2007; Boeckxstaens, 2018). While Jiang et al. (2019b) showed that the neonatal treatment of AA led to sympathetic overactivity and vagal suppression in rats, we found no changes in sympathovagal balance in our animal model induced by AA. However, consistent with their study, we demonstrated that chronic SNS could effectively alter the autonomic functions. Chronic SNS could interestingly increase vagal activity (HF) and suppress sympathetic activity (LF). The values of LF and LF/HF ratio were lower in SNS-treated rats in comparison to the baseline and sham-SNS treated rats. In addition, plasma NE (a sympathetic indicator) and PP (a marker of vagal activation) showed the same trend (Cooper et al., 2015). Consistent with the HRV results, SNS enhanced circulating PP and reduced NE. Based on previous results (Tu et al., 2020b) and the findings of this study, we speculated that the ameliorating effect on VH was mediated through the regulation of the autonomic nervous function via the CNS afferents and vagal efferents as well as local pelvic splanchnic efferents.

In recent years, VH has been linked to low-grade inflammatory involving the ENS and mast cells with direct cross-interactions on each other (De Winter et al., 2012; Karhausen et al., 2013; Jin et al., 2017; Lennon et al., 2018). VH, as one of the hallmarks of IBS associated with abdominal pain, is referred to a decreased pain threshold and/or an exaggerated response following nociceptor stimulus (Chichlowski and Rudolph, 2015). A few possible pathogeneses of this phenomenon could be (Pusceddu and Gareau, 2018) over-sensitization of primary sensory neurons, Hungin et al. (2003) hyper-excitability of spinal ascending neurons, and (Locke et al., 1997) changes in the central perception of a painful stimulus (Sengupta, 2009). Few studies have shown that mucosal afferents can become mechanically hypersensitive in chronic VH (Brierley et al., 2009; Hughes et al., 2009). Moreover, others have shown that mast cells play a key role in IBS (Adam et al., 2013; Heron and Dubayle, 2013; Zhang et al., 2016; Robles et al., 2019) and also for VH (Ohman and Simren, 2010; Zhang et al., 2016). Mast cells hyperplasia and/or overactivation has been shown to be a common feature in IBS patients (Robles et al., 2019). Vivinus-Nebot et al. (2012) reported that the severity of IBS is significantly correlated with the quantity of colonic mast cells and spontaneous release of tryptase/histamine. Furthermore, Barbara et al. (2004) showed that the close proximity of mast cells to nerves significantly correlated with the severity and frequency of abdominal pain/discomfort. Mast cells within the GI tract, communicate with extrinsic afferents to regulate nociception (Bischoff and Kramer, 2007; Boeckxstaens, 2018). Colonic mucosal biopsies from IBS patients showed an increase release of mast cells’ mediators, such as histamine, tryptase and the proinflammatory cytokine correlated with the severity and frequency of abdominal pain. These mediators have been shown to act on receptors expressed by colonic afferents to induce over-sensitization via TRPV1 and NaV1.7-dependent mechanisms (Grundy et al., 2019). The neonatal treatment of AA as an inducer of VH in rats has been shown to alter immune responses and correlate with the over-activity of mast cells in rats (Yang et al., 2019). Targeting mast cells deactivators (cromoglycate and ketotifen) and antagonists of histamine and serotonin receptors, have been tried in IBS patients in several studies, and partially exhibited good efficacy in symptom improvement (Camilleri, 2013; Boeckxstaens, 2018).

In this study, we found that SNS increased ACh and ChAT in the colon tissue, suggesting an cholinergic anti-inflammatory pathway involved in the observed analgesic effect of SNS, similar to what has been discovered in vagal nerve stimulation (Bonaz et al., 2013, 2016; Jin et al., 2017; Tu et al., 2020b). Although chronic SNS was not able to alter the number of ganglionic neurons (PGP9.5 positive cells) compared with sham-SNS in colon, it could efficiently change the level of protein expression of ChAT and nNOS. While the protein expression of ChAT was enhanced in colon tissue, nNOS showed a decrease following chronic SNS treatment. This data supported our previous findings to showing the ameliorating effect of SNS on ENS neuronal functions in a rodent model of inflammatory bowel disease (Tu et al., 2020b).

Regarding the effects of ACh on the mast cells, interestingly, mast cells have both muscarinic and nicotinic cholinergic receptors (Masini et al., 1983; Kindt et al., 2008). In spite of that, studies have shown different outcomes describing the functional effects of ACh on the mast cells. Initial investigations reported that ACh via muscarinic receptors could activate the mast cells for degranulation (Fantozzi et al., 1978; Blandina et al., 1980; Masini et al., 1985). Other investigators, however, failed to show an effect of ACh on mast cell degranulation (Kazimierczak et al., 1980; Leung and Pearce, 1984). Masini et al. (1985) demonstrated that abundance of IgE could increase the sensitivity of mast cells to ACh via muscarinic receptor and induced more degranulation. On the other hand, nicotinic receptors (nAChR) seem to mediate an anti-inflammatory effect in the presence of ACh. In fact, nicotine was shown to inhibit antigen-IgE-induced degranulation of mast cells, an effect that was mirrored by the nAChR subunit agonist, GTS-21 (Kageyama-Yahara et al., 2008). This was also shown by other investigators demonstrating a potential therapeutic effect for the cholinergic anti-inflammatory pathway in an experimental murine model of food allergy. Yamamoto et al. (2014) showed vagal stimulation by 2-deoxy-D-glucose and drug treatment with nAChR agonists could alleviate the food allergic symptoms in the gut. Previously, we showed that SNS could reduce the colonic permeability by overexpression of tight-junction’s proteins in mucosa which leads to less allergen exposure and thus IgE to mucosa (Tu et al., 2020b). Gathering with our findings that shows a downregulation in Tryptase and PGE2 expression in colon tissue, this data can support that ACh in absence of IgE might activate the nicotinic receptor to suppress the overactivation of mast cells to reduce the inflammation. On the other hand, as a mutual interaction between mast cells and neurons, it was shown that activated mast cells in rodents could release histamine/Tryptase and that can act on H3 receptor of ENS to suppress the release of ACh from enteric neurons (Frieling et al., 1993; Liu et al., 2000; Buhner and Schemann, 2012). Interestingly, our findings showed that SNS was able to suppress the mast cells activation by downregulation of Tryptase, PGE2, and cyclooxygenases-2 (COX2) and thus increase the ACh release via ENS neurons. Furthermore, it has been demonstrated in both animal models and humans that mast cell mediators in particular histamine, proteases (Tryptase) and PGE2 has substantial effects on sensitization of extrinsic afferent neurons (Jiang et al., 2011; Peiris et al., 2011; Buhner and Schemann, 2012). PGE2 is an important prostaglandin in inflammatory and immune responses (Agard et al., 2013). It was revealed that upon stimulation by various proinflammatory stimuli such as TNF-α, IL-1β, PGE2 synthesis is upregulated by the expression of COX2 (Brierley and Linden, 2014). PGE2 is an essential molecule to regulate the activation of several immune cells, particularly those involved in innate immunity such as macrophages, neutrophils, natural killer cells, and dendritic cells (Agard et al., 2013). Grabauskas et al. (2020) showed that inflammatory mediators such as protease (Tryptase) and histamine activate COX2 to increase the synthesis of PGE2 from arachnoid acid by the mast cells and furthermore, demonstrated that administration of EP2 antagonist prevents the development of VH in a rodent IBS model (Agard et al., 2013; Grabauskas et al., 2020). Alongside with this, they also revealed that this process was enhanced by Sub-P and NGF released from intrinsic sensory nerves which in turn increases the synthesis of arachnoid acid, the substrate for COX2 as well as decreasing visceral pain threshold (Jiang et al., 2019a; Grabauskas et al., 2020).

Nerve growth factor is one member of the neurotrophic factor family with an important regulatory role in the survival, growth, differentiation and function of neurons (Aloe, 2011). Moreover, NGF was shown to be associated with several pathophysiological factors of VH, such as impaired barrier function and a lowered threshold to noxious stimuli (Stanzel et al., 2008; Thin et al., 2013). The NGF expression was also shown to be up-regulated in other inflammatory diseases such as asthma (Frossard et al., 2004), arthritis (Manni and Aloe, 1998) and psoriasis (Raychaudhuri and Raychaudhuri, 2004), suggesting a role for this neurotrophin in inflammatory diseases. It was reported that the level of NGF was significantly higher in both IBS patients and animal models with VH (Tsang et al., 2012; Xu et al., 2017). Some scholars found that NGF participated in the VH process by regulating neuroplasticity and neurosynaptic transmitter secretion, which led to a decrease of visceral pain threshold (Tsang et al., 2012; Winston and Sarna, 2016). Jiang et al. (2019a) showed that SNS was able to downregulate NGF as well as tyrosine kinase A (TrkA), a high affinity receptor for NGF via MAPK/ERK pathway, PI3K/Akt pathway and PLC pathway. Sub-P and NGF as inflammatory mediators can activate specific receptors on sensory afferents, which leads to a localized membrane depolarization and change in membrane potential that can be sufficient to activate voltage-gated ion channels, leading to action potential generation and transmission to the central nervous system (Grundy et al., 2019). Interestingly, alongside with their study, we showed that chronic SNS was able to downregulate both Sub-P and NGF. The protein expression of NGF was still overexpressed at the end of the study in the sham-SNS group but normalized in the SNS-treated group. This data seemed to suggest that SNS might reduce NGF and inflammation by decreasing sympathetic innervation in the mucosal layer. As mast cells were also reported to synthesize NGF (Skaper, 2001), the reduction in the NGF expression might reflect a reduced mast cell activity with chronic SNS. *In vivo* animal studies have suggested that Sub-P is involved in the regulation of intestinal VH (Hallgren et al., 1998; Keita et al., 2010). Indeed, sub-P was found to increase mucosal permeability through producing nitric oxide and increasing inflammation (Youakim and Ahdieh, 1999; Ma et al., 2004). We found that the neonatal AA treatment increased Sub-P by more than two folds in the colon tissue, which was remarkably reduced with SNS.

There were a few limitations in this study such as the use of only male rats. Further studies are needed to investigate if SNS is able to ameliorate the VH in females as not only IBS is predominant in women, but also there are sex differences in mast cell functions. Nevertheless, this study showed that chronic SNS is able to ameliorate visceral pain in an animal model of AA-induced VH by suppressing mast cell overactivity possibly via modulation of the interaction between the enteric nervous system and mast cells, mediated by the autonomic pathway.

## Data Availability Statement

The raw data supporting the conclusions of this article will be made available by the authors, without undue reservation.

## Ethics Statement

The animal study was reviewed and approved by Nancy Athor, Director–ACUC Johns Hopkins University.

## Author Contributions

JC and JY: study concept and design, analysis and interpretation of data, critical revision of the manuscript for important intellectual content, study supervision, and obtaining funding. XJ and PG: study design, performing the research, acquisition of data, analysis and interpretation of data, drafting of the manuscript, and statistical analysis. All authors contributed to the article and approved the submitted version.

## Conflict of Interest

The authors declare that the research was conducted in the absence of any commercial or financial relationships that could be construed as a potential conflict of interest.
